# The Effect of Stromal-Derived Factor 1*α* on Osteoinduction Properties of Porous *β*-Tricalcium Phosphate Bioceramics

**DOI:** 10.1155/2021/8882355

**Published:** 2021-04-29

**Authors:** Fangchun Jin, Qixun Cai, Wei Wang, Xiaohui Fan, Xiao Lu, Ning He, Jing Ding

**Affiliations:** ^1^Department of Pediatric Orthopaedics, Xinhua Hospital, School of Medicine, Shanghai Jiao Tong University, No. 1665, Kongjiang Road, Shanghai 200092, China; ^2^Department of Orthopaedics, Shanghai No. 6th Hospital, School of Medicine, Shanghai Jiao Tong University, No. 600, Yishan Road, Shanghai 200233, China; ^3^Shanghai Key Laboratory of Advanced High Temperature Materials and Precision Forming, School of Materials Science and Engineering, Shanghai Jiao Tong University, Shanghai 200240, China; ^4^School of Materials Science and Engineering, South China University of Technology, Guangdong 510641, China; ^5^Department of Orthopaedics, Shanghai Eighth People's Hospital, No. 8 Caobao Rd., Shanghai 200235, China

## Abstract

*β*-Tricalcium phosphate (TCP) is a type of bioceramic material which is commonly used for hard tissue repair and famous of its remarkable biocompatibility and osteoconductivity with similar composition to natural bone. However, TCP lacks osteoindcutive properties. Stromal-derived factor 1*α* (SDF-1*α*) can promote bone regeneration with excellent osteoinduction effect. In this study, SDF-1*α* was loaded into TCP to investigate the *in vitro* effects of SDF-1*α* on the osteoinductive properties of TCP. *In vitro* studies showed that SDF-1*α*/TCP scaffold significantly stimulated the expression of osteopontin and osteocalcin. As to the *in vivo* studies, the rabbit bone defect model showed that SDF-1*α* stimulated more new bone formation. In conclusion, SDF-1*α*/TCP bioceramic scaffolds could further promote bone regeneration compared to pure TCP bioceramics.

## 1. Introduction

Diseases such as infection, congenital deformity, trauma, and tumor excision can result in segmental bone defects, which always lead to dysfunction and osteogenesis obstacles [1]. Reconstruction of segmental bone defects generally requires multiple surgical interventions, each procedure being associated with risks of perioperative complications [2]. Bone tissue engineering techniques that seed cells, such as bone mesenchymal stem cells (BMSCs), into scaffolds provide an optimal method to repair large bone defects [3].

Bioceramics are crucial components for bone tissue engineering applications and have been widely studied [4]. Among them, *β*-tricalcium phosphate (*β*-Ca_3_(PO_4_)_2_, TCP) is commonly used for hard tissue repair [5]. It is famous for its remarkable biocompatibility and osteoconductivity and its similarity in composition to natural bone [5]. It can also be used as scaffold for delivery of cells and growth factors. However, poor osteoinductive properties obviously limit its clinic application [6].

Cellular factors play an important role in bone regeneration, especially in bone tissue engineering [7]. Among them, stromal cell-derived factor-1*α* (SDF-1*α*) signaling pathway is well known for its osteoinductivity [8]. Moreover, SDF-1*α* can recruit migration of mesenchymal stem cells [9] and create a matrix environment conducive to cartilage and bone defect repair [10, 11]. However, loading techniques of SDF-1*α* into TCP remain a bottleneck in bone tissue engineering [12].

Taking all these aspects into consideration, combining SDF-1*α* and TCP bioceramics should be a promising path to create an osteoinductive scaffold. In this study, porous SDF-1*α*/TCP composite bioceramics were prepared to investigate the *in vitro* and *in vivo* effects of SDF-1*α* on the osteogenesis and osteoinductive properties of TCP.

## 2. Materials and Methods

### 2.1. Preparation for SDF-1*α*-Loaded TCP Porous Ceramics

Cylindrical porous TCP bioceramics (5 mm diameter and 10 mm length) were prepared as described in a previous study [6], with a homogeneous porosity of 75%, pore diameter of 500 ± 100 *μ*m, and interconnection size of 120 *μ*m. Briefly, TCP submicron powders were sieved and mechanically suspended in aqua pura. An organic skeleton made up of polymethylmethacrylate beads was impregnated with the TCP slurry. After drying, the ceramic/polymeric composite were debinded and sintered at 1100°C to obtain the porous bioceramic.

TCP were immersed in 5-time concentrated simulated body fluid at 37°C for 24 hours [13]. A dense layer of amorphous calcium phosphate that could be used as a seeding surface for the deposition of a crystalline layer was formed. The scaffolds were then immersed in a supersaturated solution of calcium phosphate containing SDF-1*α* at 37°C for 48 hours. TCP scaffolds that superficially absorbed SDF-1*α* by immersing in solution containing SDF-1*α* was used as positive control.

Before SEM characterization, the bioceramic specimens were thoroughly degreased and dried to eliminate any outgassing from organic contamination and water, then prepared by coating with a 10 nm thick Au film deposited by sputtering prior to microscopy. The microstructure of the porous samples was examined under SEM (XL-30, Phillips) with a lens detector at an accelerator voltage of 10.0 kV and different calibrated magnification levels.

### 2.2. In Vitro SDF-1*α* Release Kinetics

The release kinetics of the total amount of SDF-1*α* was assessed by measuring the extract ELISA using the SDF-1*α* ELISA Development kit under the manufacturer's instructions. Briefly, after fabrication, SDF-1*α*/TCP scaffolds were placed in PBS in 12-well plates and incubated at 37°C on a shaker table. At determined time points, the supernatant of each well was collected and replaced with fresh buffer solution. The amount of released SDF-1*α* was determined by the correlation of the measured amount to a standard curve.

### 2.3. Separation and Culture of Bone Marrow Mesenchymal Stem Cells (BMSCs)

5 ml bone marrow aspirate was collected from healthy volunteers [14], mixed with 10 ml of *α*-MEM medium (Gibco) (100 IU/ml heparinized), and then transferred to a 10 cm petri dish containing 10% fetal bovine serum (Hyclone), 1% 100 IU/ml penicillin, and 100 mg/ml streptomycin (Hyclone) and cultured in a humidified 37°C/5% CO_2_ incubator. After 3 days, nonadherent cells were washed with phosphate-buffered saline (PBS). The medium was changed every 3 days until attaining 80–90% confluence; the BMSCs were digested with 0.25% trypsin (Gibco) and subcultured.

### 2.4. Combination and Cell Culture of BMSCs on Scaffolds

The scaffolds were placed into a 24-well plate; the second passage of BMSCs was suspended at a density of 4 × 10^4^ cells/ml and loaded into TCP substrates. The cell–substrate complex was incubated at 37°C/5% CO_2_ for 2 hours. After confirmation of BMSCs being attached to the substrate, the complex was removed in another 24-well plate and cultured for 4 hours, 7 days, 14 days, and 21 days, respectively. 2 ml medium was added in every well and replaced every other day.

### 2.5. Cell Viability and Assessment

After 4 hours and 7 days of cell–substrate complex culture, the plate was washed twice with sterile PBS, half of the substrate was then transferred to a new 24-well plate, and 1 ml MTT (Sigma company) working liquid (0.5 mg/ml) was added to each well. The substrates were immersed and incubated at 37°C/5% CO_2_ for 4 hours. MTT working solution was then removed, and 1 ml isopropanol hydrochloride (0.04 N) was added to each well after the substrates were crushed. The purple crystal particles were repeatedly blown to ensure complete dissolution and then stood for 10 min. The purple solution was transferred to an EP tube and centrifuged at 12000 × g for 5 min to remove the TCP powder. The supernatant was used to measure the light absorption value on the spectrophotometer, with the wavelength of 570 nm and the reference wavelength of 650 nm. The fragments of the carrier were weighed after drying at 50°C, and the cell activity was expressed as light absorption value per gram of the carrier.

### 2.6. Detection of Osteopontin

At 7, 14, and 21 days of culture, the culture medium was collected, stored at -70°C, dissolved at room temperature, and detected by anti-human ELISA kit. Standard solution or sample 50 *μ*l was added to 96-well plates. 100 *μ*l analytical solution was added to each well and incubated at room temperature for 2 hours. Each well was washed 4 times with buffer solution. 200 *μ*l antibody containing horseradish peroxidase was added and incubated for 2 hours at room temperature. The content of each well was then removed and washed with buffer solution for 4 times; 200 *μ*l substrate was then added and incubated at room temperature away from light for 30 minutes. The absorbance of 450 nm wavelength was determined by adding 50 *μ*l termination solution to each well, and the corrected wavelength was 570 nm. The results were compared with the standard solution of human osteopontin.

### 2.7. Detection of Osteocalcin

At 7, 14, and 21 days of culture, the culture medium was collected, stored at -70°C, dissolved at room temperature, and detected by anti-human ELISA kit. 25 *μ*l of standard solution or sample was added to 96-well plates. 100 *μ*l antibody containing horseradish peroxidase was added to each well and incubated in a horizontal oscillator at room temperature for 2 hours. Each well was washed for 3 times, 100 *μ*l chromogenic agent was then added, placed on a horizontal oscillator, and incubated at room temperature for 30 minutes. The absorbance of 450 nm wavelength was detected by adding 200 *μ*l termination solution to each well, and the corrected wavelength was 650 nm. The results were compared with the standard solution of human osteocalcin.

### 2.8. Animal Experiments

**Figure 1:**
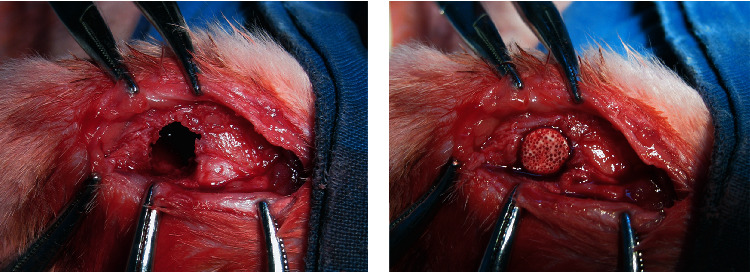
The bone defect model was established in the femoral condyle parallel of the New Zealand rabbits, and the bone cavity was filled with porous SDF-1*α*/TCP bioceramic.

The animal experiments were carried out according to the policies of Shanghai Jiao Tong University School of Medicine and the National Institutes of Health. In brief, 3-month-old New Zealand rabbits weighing 2.5 ± 0.3 kg were randomly divided into three groups. For the duration of the experiment, rabbits were housed individually in cages and provided free access to water and fed a commercial pellet diet. Six animals were used per material. The scaffolds were sterilized by gamma radiation before implantation. After administering anesthesia by 0.5 mg/kg of acepromazine (Calmivet–Vetoquinol) and 10 mg/kg of ketamine, rabbits were operated in rigorous aseptic conditions using the lateral knee approach. First, a femoral condylar cavity paralleling with the joint surface of 5 mm in diameter and 10 mm in depth was drilled at the point 1 cm above the femoral condyle, and the cortical bone window was removed with scissors. For both the SDF-1*α*/TCP composite and TCP bioceramic groups, the scaffolds were then inserted in the cavity, as is shown in Figure 1. The cavity was left unfilled in the blank control group.

### 2.9. Histological Preparation and Analysis

The samples were harvested and observed 24 weeks after implantation. All rabbits were sacrificed, and the femoral condyles were extracted. The samples were fixed in 10% formaldehyde solution buffered with PBS (pH 7.3) for 2 weeks and then rinsed under tap water for 12 h. Gradient alcohol concentration (70–100%) was used in the dehydration process for 24 h. The samples were then embedded in methylmethacrylate without decalcification. The cross sections were cut to about 200 *μ*m thickness with a Leitz Saw Microtome 1600 (Wetzlar, Germany), ground, and polished to about 50 *μ*m thickness with an Exakt Grinder (Norderstedt, Germany). Finally, the samples were stained with Van Gieson's picrofuchsin stain (V-G stain).

### 2.10. Histomorphometric Analysis

New bone volume (NBV) was measured on two sections for five fields per V-G-stained section with the help of an eyepiece micrometer (KPL 16, Carl Zeiss, Germany) and an ocular integrator with 100 points (KPL 8, Zeiss, Germany). The results were studied in two sections per sample using light microscopy [15]. New bone volume (%) represents the percentage of implant occupied by new bone tissue.

### 2.11. Statistical Analysis

The data in this study were analyzed using SPSS11.0 statistical software (SPSS Inc., Chicago, IL). The results were expressed as mean ± standard deviation, the variable data were analyzed by one-way ANOVA, and *p* < 0.05 was considered significant.

## 3. Results

### 3.1. Characterization of the Composites

**Figure 2:**
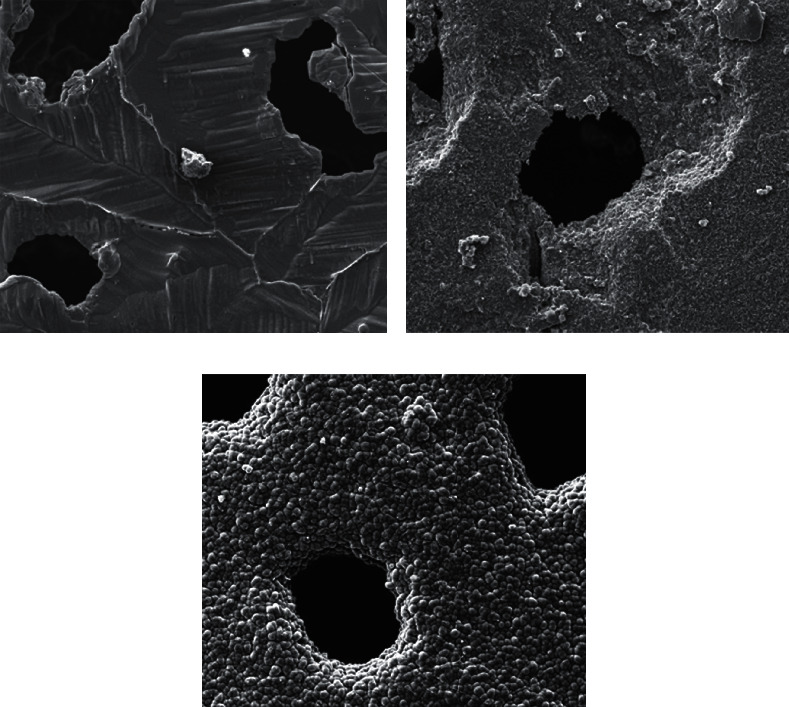
Electron microscopic view of the surface of the porous TCP bioceramics: (a) without pretreatment, the surface is smooth and not suitable for loading drugs; (b) after pretreatment, the surface becomes rough and can do well in drug loading; (c) TCP loaded with SDF-1*α* and Ca-P deposition.

According to the previous studies, bioceramics have a homogeneous pore size of 500 ± 100 *μ*m, an interconnection diameter of 120 *μ*m, and a uniform porosity of 75%. As is shown in Figure 2, observed under an electron microscope, the surface of TCP without pretreatment is too smooth for drugs or cell factors to load. It becomes suitable for drug loading after surface roughening. The SDF-1*α*/TCP composite exhibited looser and rougher surfaces with more irregular micropores and larger crystals than TCP bioceramic scaffolds, which corresponds to the results of the specific surface of TCP.

### 3.2. In Vitro SDF-1*α* Release Kinetics

**Figure 3:**
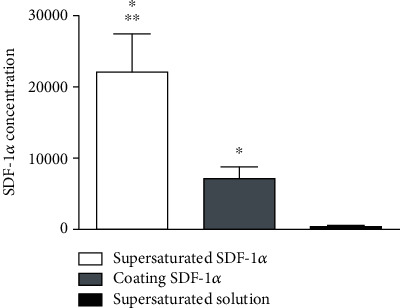
SDF-1*α* concentration in different preparation methods (^∗^VS supersaturated solution, *p* < 0.05; ^∗∗^VS coating SDF-1*α*, *p* < 0.05).

The total amount and released concentration of SDF-1*α* have a positive correlation with the loading method (supersaturated or coated), as is shown in Figure 3. The amount of SDF-1*α* released from TCP with supersaturated SDF-1*α* liquid is higher than that from SDF-1*α*-coated TCP. Almost no SDF-1*α* can be detected in supersaturated solution.

### 3.3. In Vitro Cell Viability and Osteogenic Assessment

**Figure 4:**
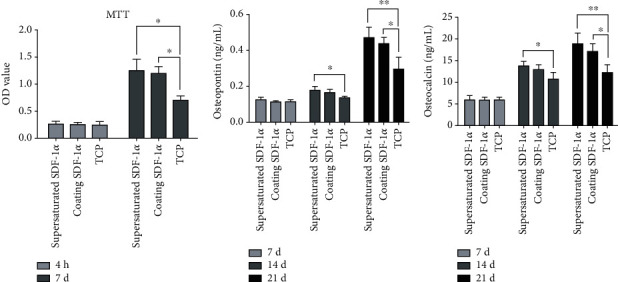
Comparison of different SDF-1*α*/TCP scaffolds on each time point. (a) OD volume showed after 7 days; cell viability in supersaturated SDF-1*α*/TCP was highest. (b) Osteopontin and (c) osteocalcin raised as time expansion, at each time point; the supersaturated SDF-1*α*/TCP group had more protein expression (^∗^*p* < 0.05, ^∗∗^*p* < 0.01).

TCP scaffold with supersaturated SDF showed the highest cell viability as well as more expression of osteopontin and osteocalcin. TCP scaffold with coating SDF-1*α* took the second place. Cell viability increased with time and was significantly higher in SDF-1*α*/TCP composites. The expression of OP and OC showed no significant difference between the TCP group and the other two groups on day 7 but was obviously lower than the others on day 14 and day 21, as is shown in Figure 4.

### 3.4. Histological Analysis

**Figure 5:**
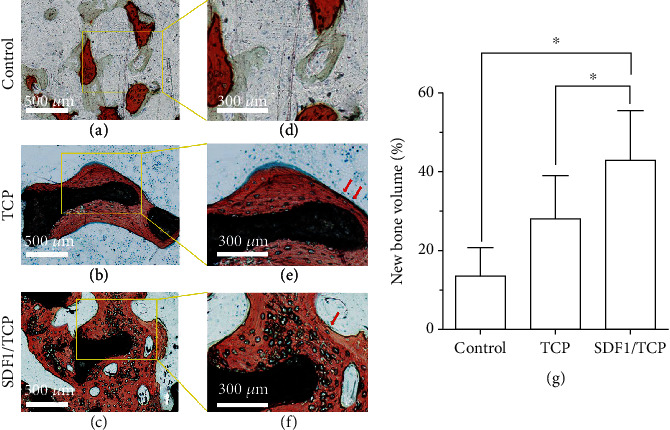
The newly formed bone (NB), osteoid tissue (OT), and residual material (M) are indicated as red, green, and black, respectively. Compared with the other two groups, the SDF-1*α*/TCP group got more volume of new bone. Red arrows indicate the lining cells and osteoblastic cells (^∗^*p* < 0.05).

Newly formed bone, osteoid tissue, and residual material are, respectively, stained red, green, and black in V-G staining. After 24 weeks of implantation, newly formed bone, rich in osteocyte lacunae, was observed on the surface of all residual implants. The lining cells and osteoblastic cells are, respectively, indicated in Figures 5(e) and 5(f) to reflect the main actors of new bone formation. Furthermore, osteoblastic cells can be detected on the new bone in the SDF-1*α*/TCP group. Compared with the other two groups, the SDF-1*α*/TCP group has more volume of new bone, as is shown in Figure 5. No cellular dysplasia was observed on the surface of any of the materials or in their neighboring areas, and no acute inflammation appeared at the interface of the implants and surrounding tissue.

## 4. Discussion

Bone scaffolds are a major tool in bone tissue engineering. In this study, SDF-1*α*/TCP composite bioceramics were prepared to investigate the effects of SDF-1*α* on the osteoinductive properties of TCP. Our *in vitro* study showed that SDF-1*α*/TCP scaffolds significantly stimulated the expression of osteopontin and osteocalcin. Meanwhile, in the *in vivo* study, the rabbit bone defect model showed that SDF-1*α* stimulated more new bone formation. Thereafter, SDF-1*α*/TCP bioceramic scaffolds could promote bone regeneration and may be an effective way to treat bone defects.

SDF-1*α* is highly expressed in bone marrow stromal cells, but it is also widely expressed in diverse organs including the brain, heart, liver, and lungs. It has been suggested that SDF-1*α* is involved in the recruitment of stem cells that differentiate into the cells necessary to repair tissue damage [16, 17]; the function may relate with AKT and Wnt/*β*-catenin signaling pathway [18, 19]. Further study indicated that DLX2 mediates Wnt/*β*-catenin signal to promote osteogenic differentiation of BMSCs [20]. A study also showed that TCP implanted in dog mandibles can increase the expression of SDF-1*α* [21], thus enhancing bone healing processes and stimulating the coordinated actions of osteoblasts and osteoclasts, leading to bone regeneration. In this study, SDF-1*α* was incorporated into TCP by a bioactive loading technique [13]. Based on the release test, SDF-1*α* was released from SDF-1*α*/TCP, which indicated that the osteoinductive properties of SDF-1*α* can be given to TCP bioceramics. The osteoinductive properties of SDF-1*α*/TCP is also proved by elevated levels of osteopontin and osteocalcin, but the signaling pathway should be investigated during further study.

The ideal biomaterial candidate for bone regeneration should be resorbable and gradually replaced by the newly formed bone. Based on previous studies of TCP, it has been shown to be gradually replaced by the newly formed bone as a process of osteoconduction [22]. However, despite the osteoconductive properties of biomaterials in bone tissues engineering, the implanted materials should also have osteoinductive properties. Various cellular factors have been used to promote the osteoconductive properties of TCP, but there is a great need of more cellular factors, especially with broad cellular effect. In our study, SDF-1*α* was loaded into TCP to give osteoinductive properties to TCP. As result, the rabbit bone defect model showed that SDF-1*α* stimulated more new bone formation although cell-free SDF-1*α*/TCP scaffolds were implanted. This may be because SDF-1*α* can recruit migration of mesenchymal stem cells [23] and create a matrix environment inductive for bone defect repair [24, 25], but further studies should be performed.

Nevertheless, this study is limited in the number of animal model and the deep mechanism of SDF-1*α*/TCP in promoting bone regeneration needs further study. Additional micro-CT analysis of the newly formed bone would also provide more data. Therefore, clinic application of such SDF-1*α*/TCP bioceramics would require further studies about its biological safety.

## 5. Conclusion

In conclusion, the addition of SDF-1*α* into porous TCP bioceramics resulted in a higher cell viability and osteoinduction. *In vitro* and *in vivo* experiments proved that SDF-1*α* had a positive influence on osteogenesis compared to pure TCP. Thus, the porous SDF-1*α*/TCP bioceramics could be a promising new way to treat bone defects instead of traditional synthetic bone grafts.

## Data Availability

All underlying data is available on request through email address of corresponding authors.
